# RETRACTED: Mohanty et al. Formulation and Optimization of Alogliptin-Loaded Polymeric Nanoparticles: In Vitro to In Vivo Assessment. *Molecules* 2022, *27*, 4470

**DOI:** 10.3390/molecules31142475

**Published:** 2026-07-15

**Authors:** Dibyalochan Mohanty, Sadaf Jamal Gilani, Ameeduzzafar Zafar, Syed Sarim Imam, Ladi Alik Kumar, Mohammed Muqtader Ahmed, Mohammed Asadullah Jahangir, Vasudha Bakshi, Wasim Ahmad, Eyman Mohamed Eltayib

**Affiliations:** 1 Department of Pharmaceutics, School of Pharmacy, Anurag University, Hyderabad 500088, India; vasudhapharmacy@cvsr.ac.in; 2 Department of Basic Health Sciences, Princess Nourah Bint Abdulrahman University, Riyadh 11671, Saudi Arabia; sjglani@pnu.edu.sa; 3 Department of Pharmaceutics, College of Pharmacy, Jouf University, Sakaka 72341, Saudi Arabia; emeahmed@ju.edu.sa; 4 Department of Pharmaceutics, College of Pharmacy, King Saud University, Riyadh 11451, Saudi Arabia; 5 School of Pharmacy and Life Sciences, Centurion University of Technology and Management, Khurda 752050, India; alikkumar3@gmail.com; 6 Department of Pharmaceutics, College of Pharmacy, Prince Sattam Bin Abdulaziz University, Al-Kharj 11942, Saudi Arabia; mo.ahmed@psau.edu.sa; 7 Department of Pharmaceutics, Nibha Institute of Pharmaceutical Sciences, Rajgir 803116, India; asadullahjahangir@gmail.com; 8 Department of Pharmacy, Mohammed Al-Mana College for Medical Sciences, Dammam 34222, Saudi Arabia; wasima@machs.edu.sa

The journal retracts the article titled “Formulation and Optimization of Alogliptin-Loaded Polymeric Nanoparticles: In Vitro to In Vivo Assessment” [1], cited above.

Following the publication, concerns were brought to the attention of the Editorial Office regarding the scientific accuracy of the data presented in the published paper [1].

In accordance with the journal’s standard procedures, the Editorial Office and Editorial Board conducted an investigation, which confirmed inconsistencies within the data presented in Figure 7. Although the authors cooperated with the investigation, the explanations and supporting materials provided were not deemed sufficient by the Editorial Board to resolve the identified concerns. As a consequence, the Editorial Board has lost confidence in the reliability of the reported findings and has decided to retract this publication in accordance with MDPI’s retraction policy.

This retraction was approved by the Editor-in-Chief of the *Molecules* journal.

Author E.E. agreed to this retraction. Author D.M. did not agree to this retraction. The remaining authors did not respond to correspondence regarding this retraction.
